# Supplementary material for: The North Equatorial Current and rapid intensification of super typhoons
Source: Nat Commun. 2024 Mar 7;15:1742. doi: 10.1038/s41467-024-45685-2 (PMC10920791; doi:10.1038/s41467-024-45685-2)
Supplement: Supplementary file 1 — Supplementary Information [file 41467_2024_45685_MOESM1_ESM.pdf]

Supplementary Information

Supplementary Figures

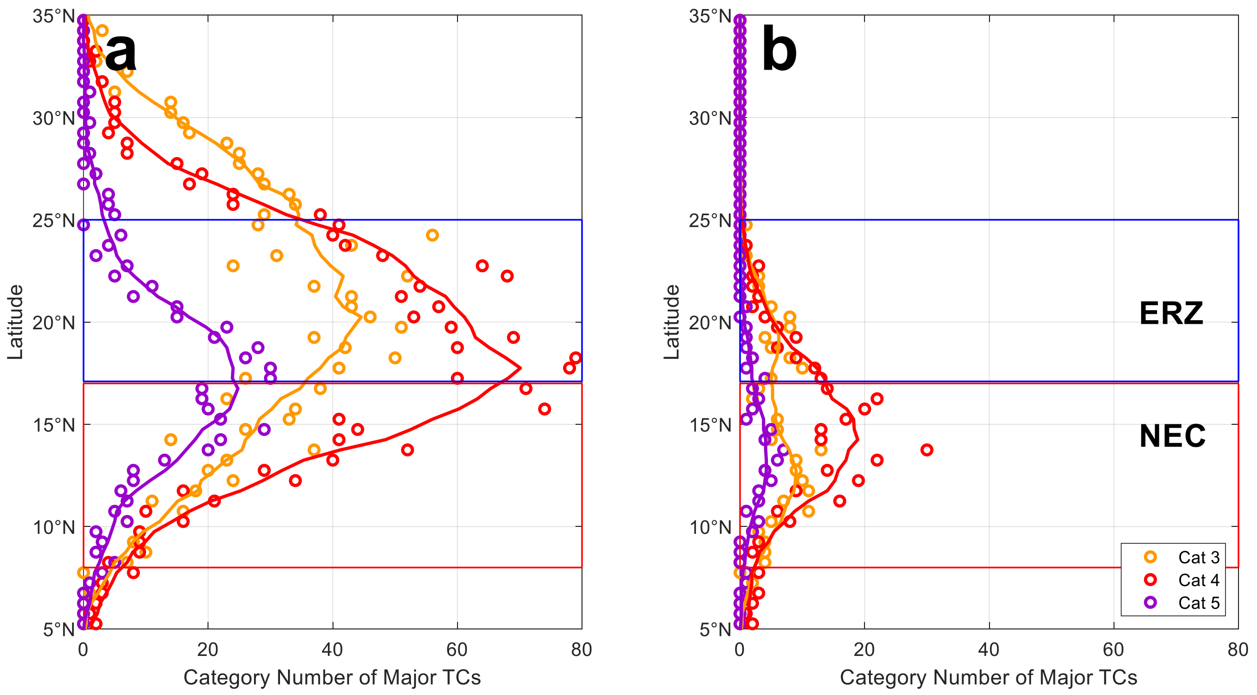

Supplementary Fig. 1 Meridional distribution of the accumulated occurrence (in days) of major TCs (CAT3 to CAT5) in the western North Pacific (120°–160° E). For (**a**) all seasons and (**b**) off-TC seasons (December–April) from the JTWC data for 1984–2021. Colored open circles are 0.5°-averaged values, with overlaid continuous curves representing a smoothing over 1˚ latitude.

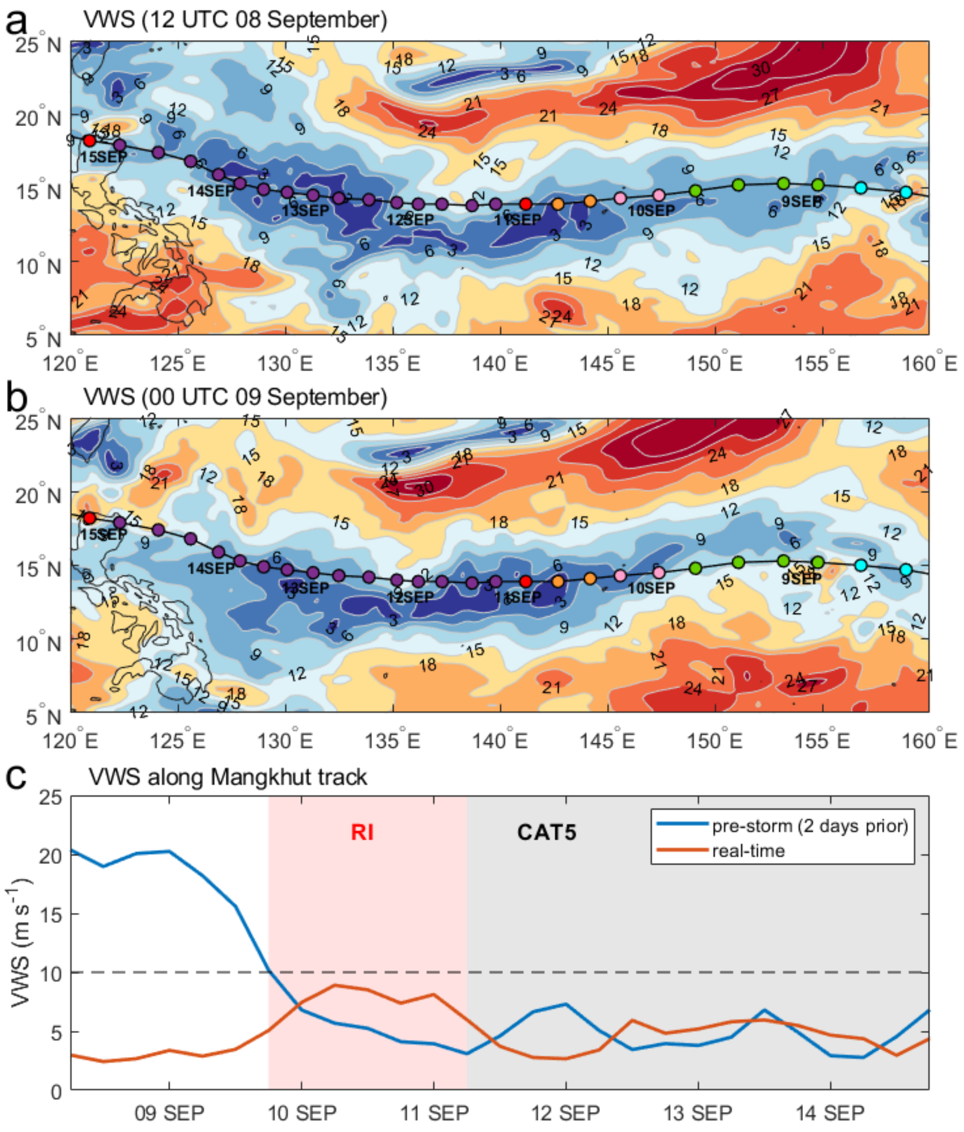

Supplementary Fig. 2 Atmospheric impact on TC intensification. a–b, Spatial distributions of VWS (shading in m s^{-1}) at (**a**) 12 UTC 08 and (**b)** 00 UTC 09 September 2018 before Mangkhut reaching its rapid intensification. (**c**) Temporal evolution of pre-storm (blue line, 2-days prior) and real-time VWS (red line) averaged within a radius of 200 km with regard to the typhoon center by using the ERA5 during the period of 12 UTC 08 – 18 UTC 14 September 2018. Real-time VWS was calculated by removing typhoon using the Kurihara filter[S1, S2].

The environmental VWS can suppress the TC intensification with a critical value greater than 10 m s^{-1} [31–35]. Ref.[35] demonstrated that RI could occur under the moderate VWS of 5 to10 m s^{-1}.

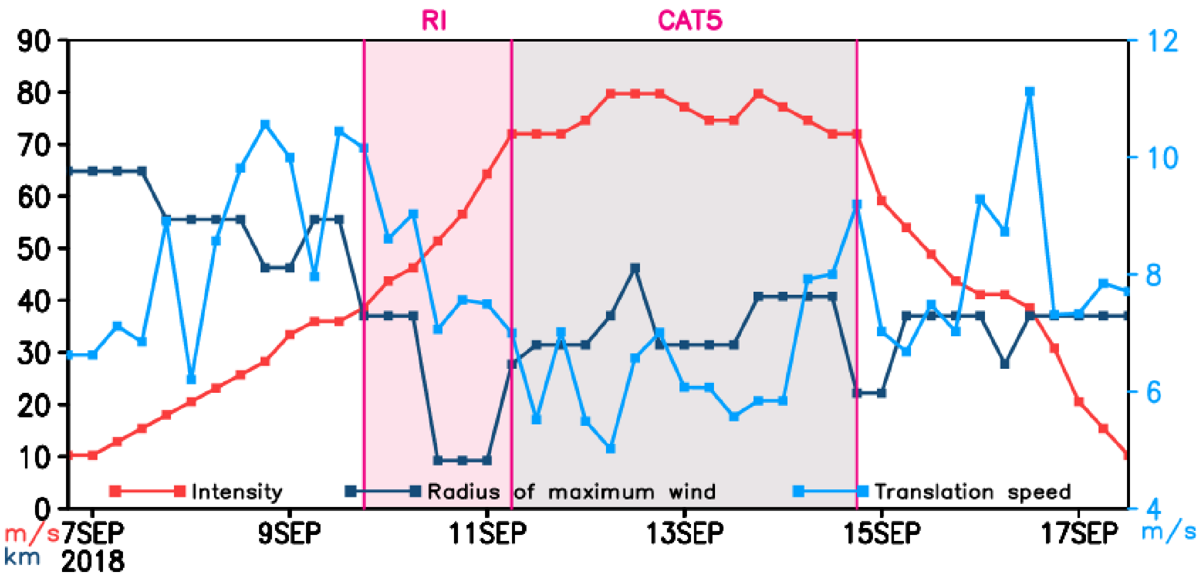

Supplementary Fig. 3 Super typhoon Mangkhut activity. Intensity (red line, in m s^{-1}), radius of maximum wind (dark blue line, in km), and translation speed (blue line, in m s^{-1}).

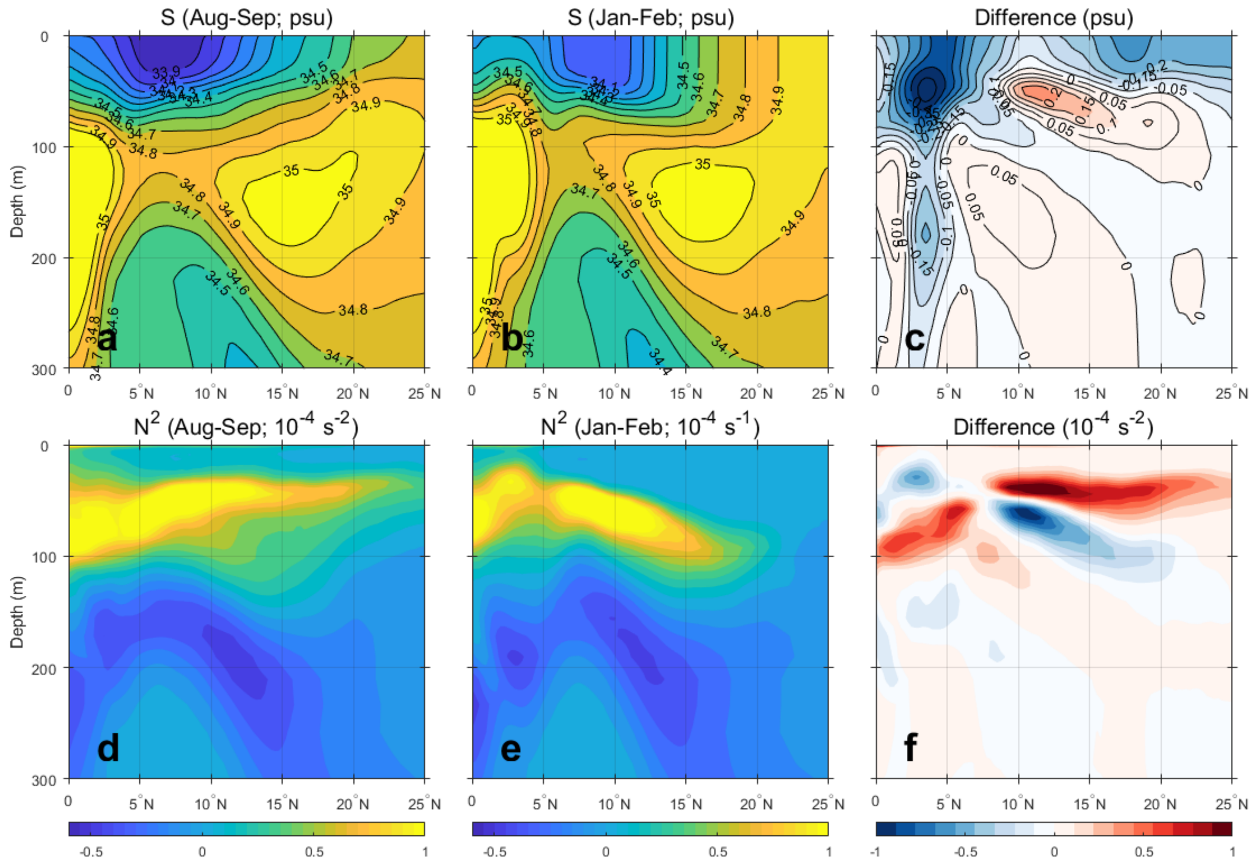

Supplementary Fig. 4 Climatological mean vertical salinity and salinity-induced squared buoyancy frequency N^2 sections across the NEC region. Longitudinal-mean (123°–160°E) meridional sections of (**a**) summer mean (August-September) salinity, (**b**) winter mean (January-February) salinity, and (**c**) difference (AS-JF), using ORAS5. Salinity-induced squared buoyancy frequency N^2 sections of (**d**) summer mean and (**e**) winter mean, and (**f**) difference (AS-JF). Climatological data for salinity is from ORAS5 (Methods).

During the summer typhoon season (August-September), the northward migration of the ITCZ (Fig. 4a-c) results in a substantial influx of fresh water into the surface layer of the NEC region (Supplementary Figs. 4a, c). As a result, salinity difference between summer and winter (January-February) reveals a clear summer freshening in the surface layer of about 50-m thick over the whole NEC region (Supplementary Fig. 4c), although the seasonal difference in freshening appears small at 10-13°N in the core region of ITCZ. The seasonal migration of ITCZ contributes to a latitudinal broadening of fresh water in the surface layer over the whole western North Pacific, with a maximum in salinity-induced N^2 difference during the TC peak season, August-September (Fig. 4f).

Also note that salinity-driven high N^2 or enhanced stratification in the NEC region is much shallower (30-50 m) in summer (Supplementary Fig. 4d) than that in winter (50-100 m) (Supplementary Fig. 4e). The seasonal difference in stratification (Supplementary Fig. 4f) indicates that the enhanced stratification at the subsurface layer at about 30-50m depth during the TC peak season should result from the enhanced fresh water influx from the ITCZ. This enhanced salinity-driven stratification (red layer at 30-50m in Supplementary Fig. 4f) eventually leads to weakening TC-induced mixing over the NEC latitudes. In effect, salinity-driven cooling inhibition CI increases by about 7.5-12.5% over the NEC region (Supplementary Fig. 6). This freshening process may explain reducing surface cooling by 19% for Super Typhoon Mangkhut, according to the Price-Weller-Pinkel model (Supplementary Fig. 5).

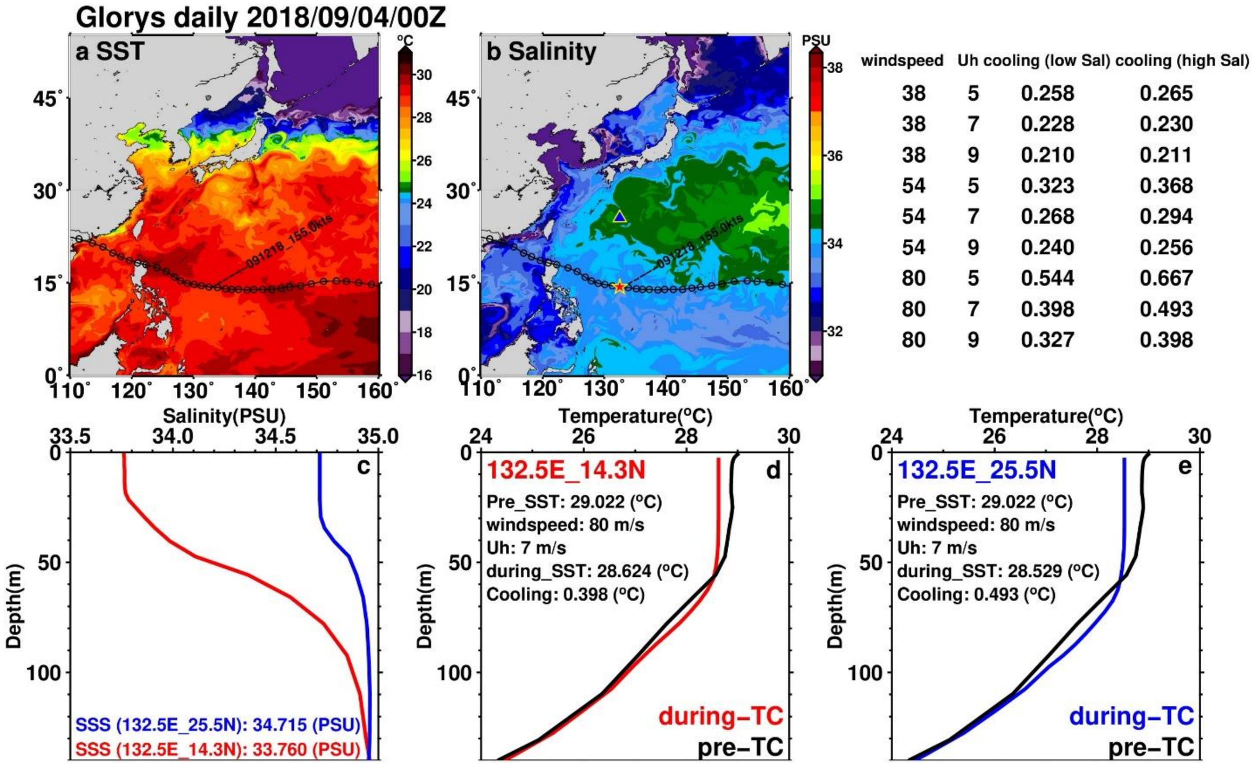

windspeed	Uh	cooling (low Sal)	cooling (high Sal)
38	5	0.258	0.265
38	7	0.228	0.230
38	9	0.210	0.211
54	5	0.323	0.368
54	7	0.268	0.294
54	9	0.240	0.256
80	5	0.544	0.667
80	7	0.398	0.493
80	9	0.327	0.398

Supplementary Fig. 5 3D Price-Weller-Pinkel numerical experiments comparing low-salinity vs. regular salinity conditions. (**a**) daily Sea Surface Temperature (SST) and (**b**) daily Sea Surface Salinity (SSS) on Sep.04, 2018, for pre-TC, before Super Typhoon Mangkhut arrives. (**c**) salinity profiles at points of ERZ (blue, regular 'high salty' condition, location shown as triangle in (b)) and NEC (red, low salinity condition, where Mangkhut passed, location shown as star in (b)). (**d**, **e**) TC-induced cooling results, where the black profile is the pre-TC temperature profile.

The experiment has been carried out using an identical temperature profile but two different salinity profiles, one along Mangkhut's track (the fresh scenario, see Supplementary Fig.5c, red line) and the other at the ERZ (the regular 'salty' scenario, see Supplementary Fig.5c, blue line).

Since the same pre-TC temperature is used, the difference between the two experiments are only due to the pre-TC salinity profiles. The low-salinity results (d) shows weaker cooling as compared to the regular high-salinity result in (e). The wind forcing is based on the observed Mangkhut (80 m $^{-1}$) and the observed translation speed of 7 m s^{-1} during the Mangkhut period. Additional results based on different wind speeds and translation speeds are shown in the upper right table of the figure. Consistently, the TC-induced cooling effect in the low-salinity group is weaker than in the high-salinity group for all wind speeds and translation speeds.

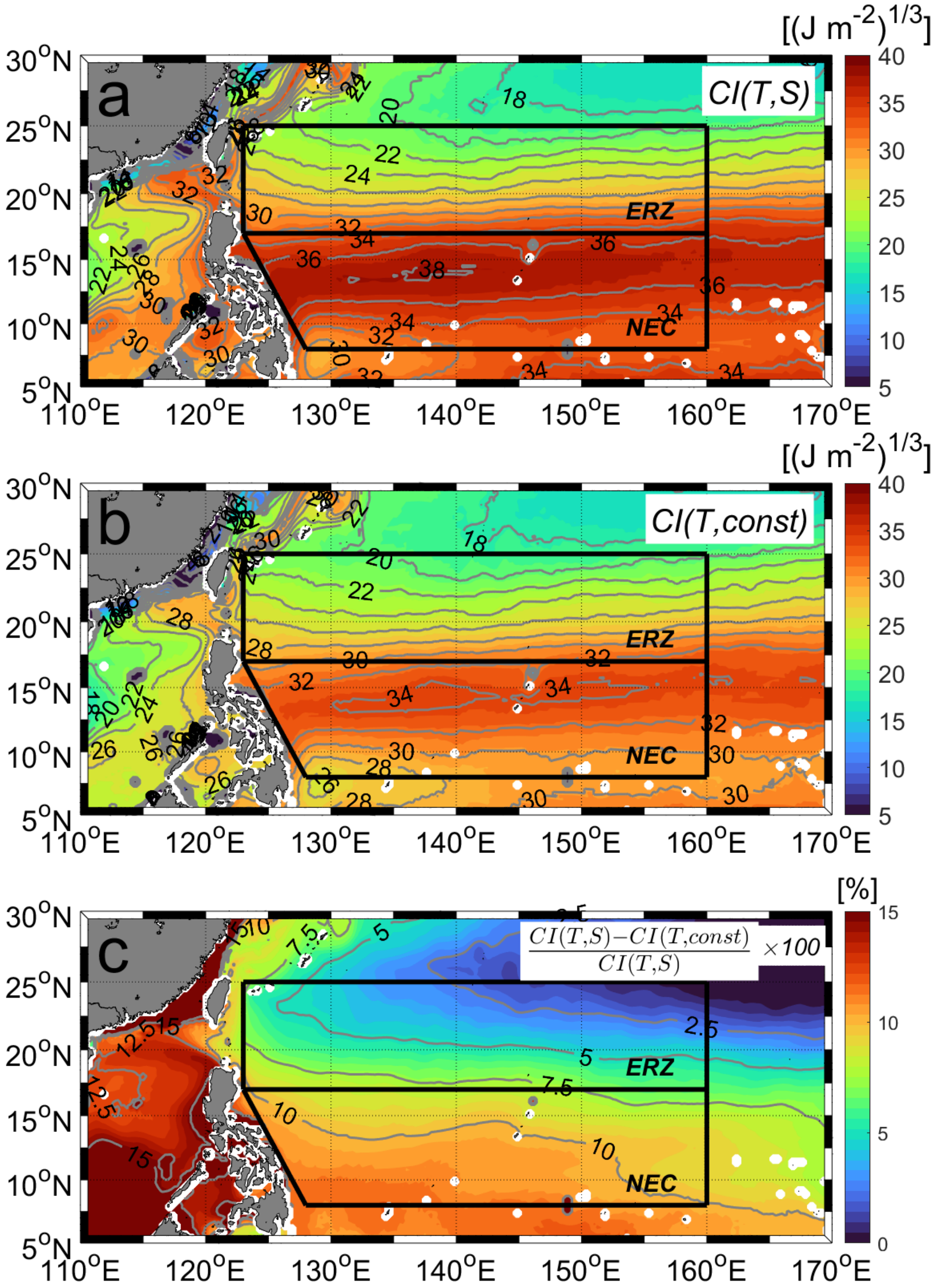

Supplementary Fig. 6 Cooling inhibition index (CI) and CI difference with constant salinity. (**a**) CI (T, S) (Methods) from season mean (June–November) temperature and salinity using climatological mean data from the Ocean Reanalysis System 5 data (ORAS5) and (**b**) CI (T, 33) with a constant salinity of 33 psu, and (**c**) 100*(CI(T, S) - CI(T, const))/CI(T, S) (%).

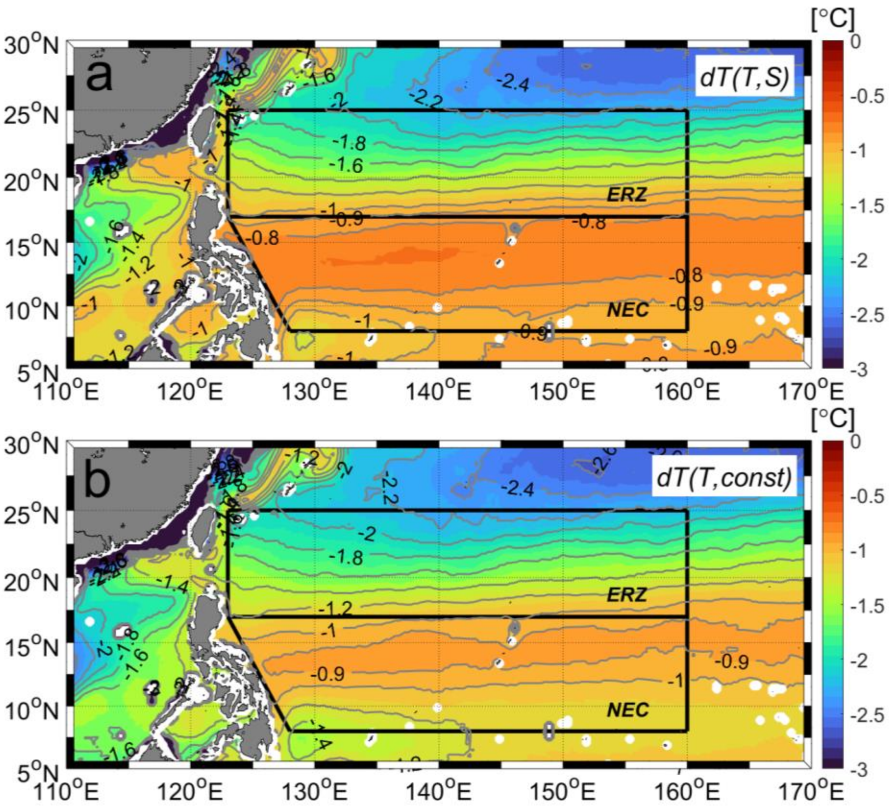

Supplementary Fig. 7 TC-induced cooling d*T* (°C) computed from Vincent et al., (2012) [25] climatology for CAT5 super typhoon translating at 6-7 m s^{-1}, which is a function of cooling inhibition index CI. (a) SST cooling d*T* (WPi, CI)[25] diagnosed for wind power index WPi = 4.1, which corresponds to CAT5 super typhoon with a translation speed of 6-7 m s^{-1}, and cooling inhibition index CI computed using climatological temperature and salinity profiles in summer mean (June-November) from ORAS5. (**b**) same as (a) but for constant salinity of 34 psu, CI (*T*, *S*=34). The CI fields are shown in Supplementary Fig. 6.

CAT5 super typhoon with a translation speed of 6-7m s^{-1} corresponds to wind power index WPi = 4.1. (Fig.4b of ref. [25]). For fixed WPi = 4.1, TC-induced cooling d*T* is a function of cooling inhibition index CI only (Fig.7b of ref. [25]) (Methods). Two different maps are constructed: one corresponding to the CI (*T*, *S*) field computed from seasonal mean (June–November) temperature and salinity profiles from ORAS5, and the other for constant salinity, CI (*T*, *S*=34) (Supplementary Fig.7). The difference of two cooling maps may indicate the barrier layer effect due to the fresh water inflow from the rainy ITCZ, which contributes significantly (up to 15%) to cooling inhibition in the NEC region. According to the observational results from Mangkhut (Fig. 2e), we regard 0.9°C as the upper limit of cooling (except one outlier close to 1°C) for the existence of CAT5 super typhoon in the NEC region, as can be seen in Supplementary Fig. 7a.

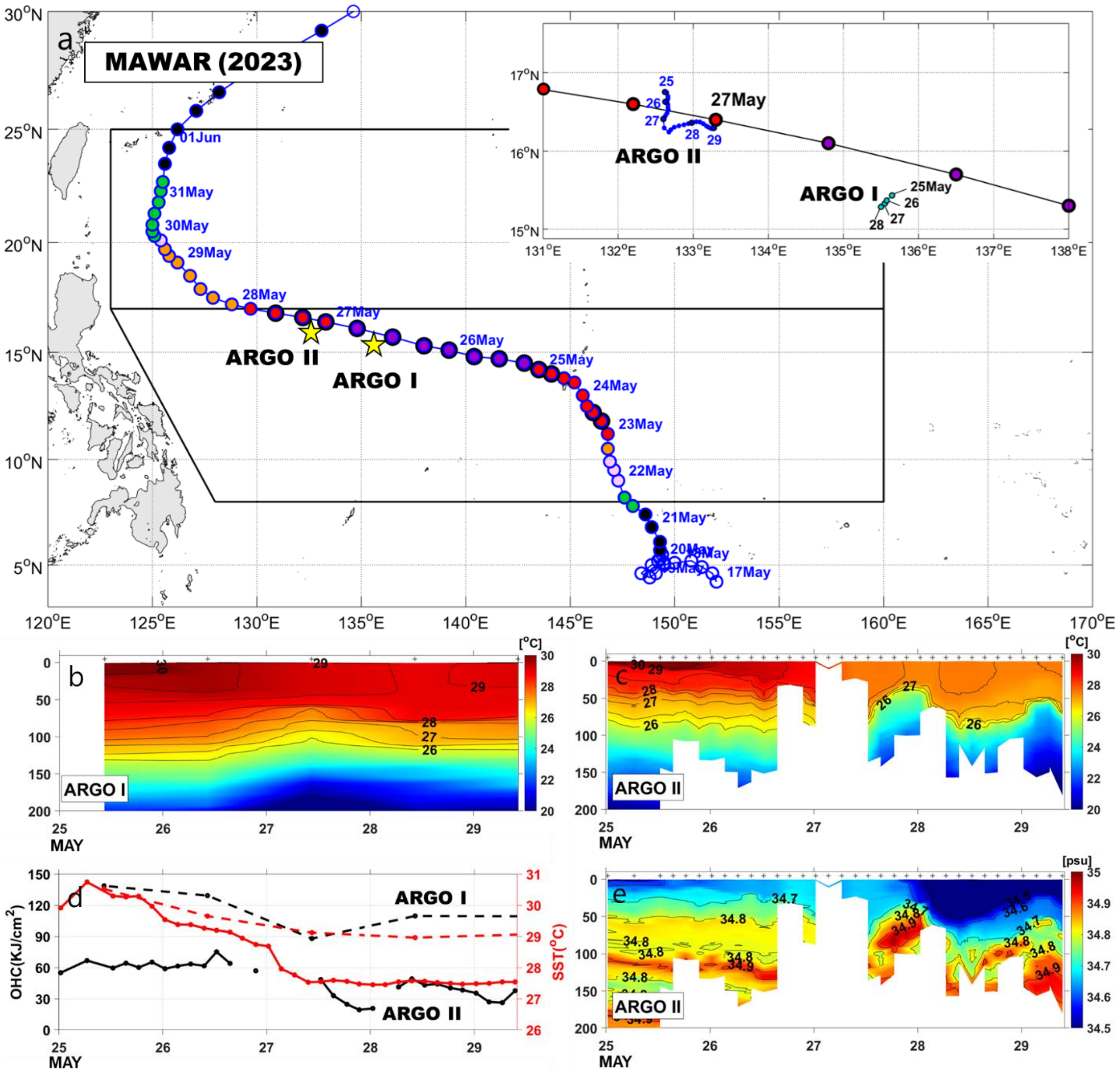

Supplementary Fig. 8 (a) Track of Mawar from 17 May to 2 June 2023 from the JTWC best track data. The colored closed circles represent the TC intensity using the same scale as in Fig. 1a. The approximate position of two floats (ARGO I, II) data (Methods) available in the area within 100 km from the Mawar track is indicated by stars, with their inserted zoomed position. (**b**) Depth-time section of temperature for ARGO I. (**c**, **e**) Depth-time sections of temperature and salinity for ARGO II. (**d**) Time series of SST (red) and OHC (black) for ARGO I (dotted lines) and ARGO II (solid lines).

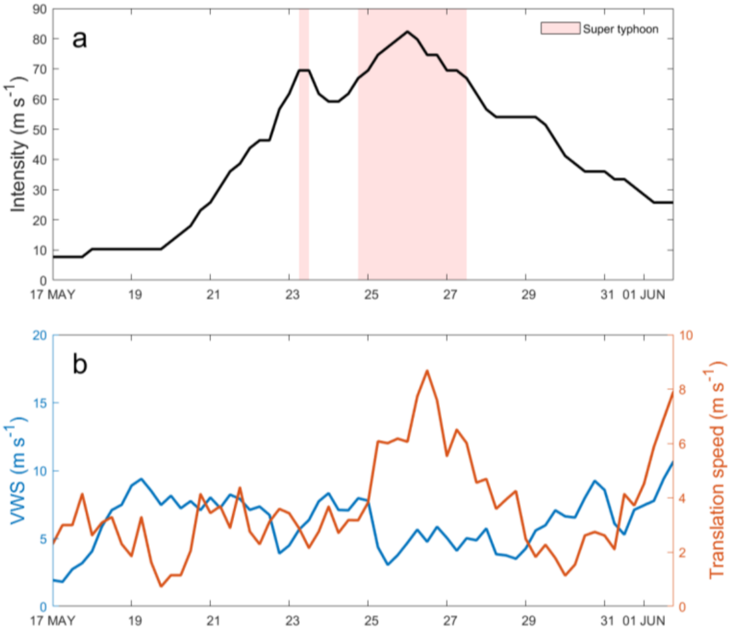

Supplementary Fig. 9 (**a**) JTWC intensity of Mawar in the western North Pacific, where it kept as super typhoon in the NEC region (Supplementary Fig. 8a). (**b**) Vertical wind shear (VWS, blue) and translation speed (red) of Mawar, showing favorable atmospheric conditions (a high translation speed >5 m s^{-1} and a low VWS <5 m s^{-1}) during its super typhoon stage (25-27 May) in the NEC region. Mawar was CAT5 from 06:00 25th to 18:00 26th May.

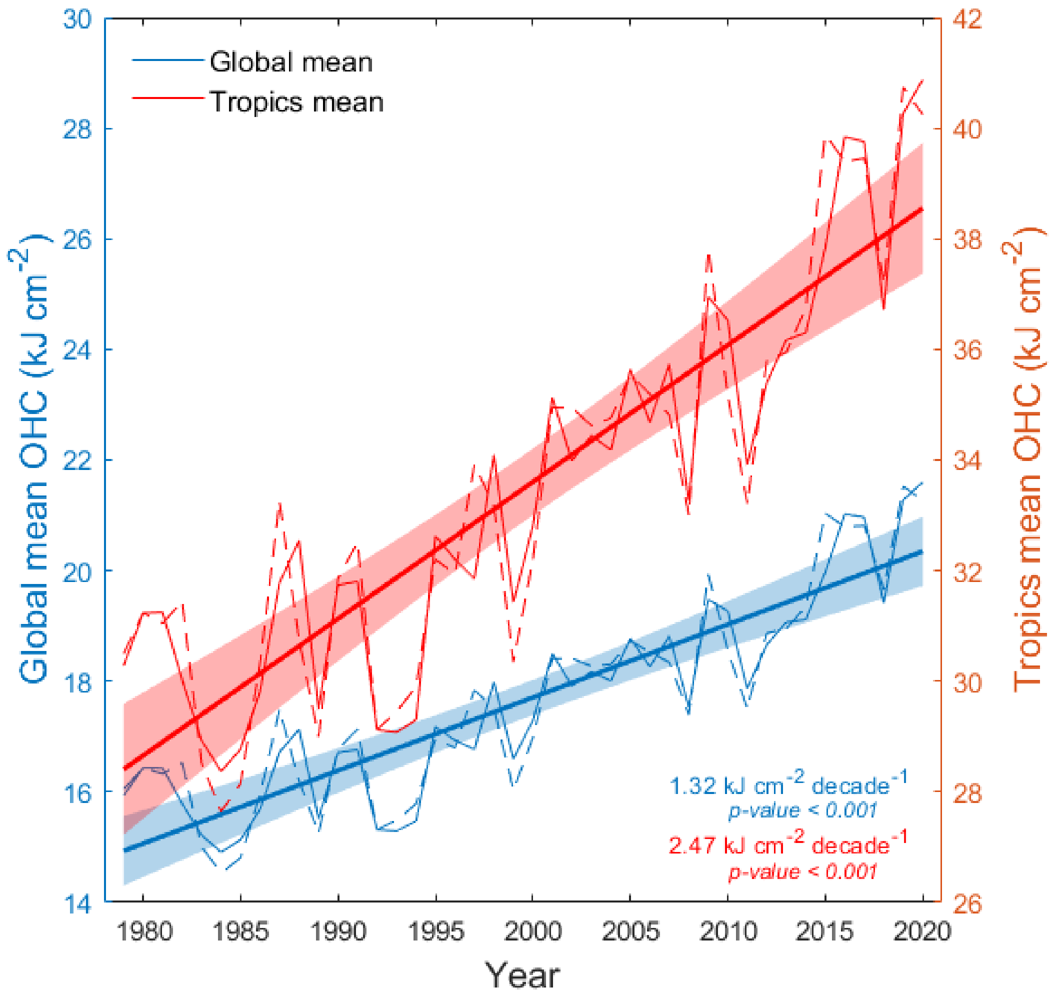

Supplementary Fig. 10 Time series of seasonal mean OHC in the global (blue lines) and tropics regions (red line, 30°S-30°N). The solid lines represent OHC time series without ENSO signals, which have previously been removed by subtracting the ENSO-regressed time series from the original time series (discontinuous line). The shaded area indicates a 95% confidence interval. OHC computed from the season-mean (June–November) ORAS5 data.

Supplementary References

S1. Kurihara, Y., Bender, M. A. & Ross, R. J. An initialization scheme of hurricane models by vortex specification. *Mon. Wea. Rev.* **121**, 2030–2045 (1993).

S2. Kurihara, Y., Bender, M. A., Tuleya, R. E. & Ross, R. J. Improvements in the GFDL hurricane prediction system. *Mon. Wea. Rev.* **123**, 2791–2801 (1995).
